# Increase in periosteal angiogenesis through heat shock conditioning

**DOI:** 10.1186/1746-160X-7-22

**Published:** 2011-11-18

**Authors:** Majeed Rana, Constantin von See, Martin Rücker, Paul Schumann, Harald Essig, Horst Kokemüller, Daniel Lindhorst, Nils-Claudius Gellrich

**Affiliations:** 1 Department of Oral and Maxillofacial Surgery, Hannover Medical School, Hannover, Germany

**Keywords:** Heat shock, periosteum, animal, intravital microscopy, calvaria, microcirculation

## Abstract

**Objective:**

It is widely known that stress conditioning can protect microcirculation and induce the release of vasoactive factors for a period of several hours. Little, however, is known about the long-term effects of stress conditioning on microcirculation, especially on the microcirculation of the periosteum of the calvaria. For this reason, we used intravital fluorescence microscopy to investigate the effects of heat shock priming on the microcirculation of the periosteum over a period of several days.

**Methods:**

Fifty-two Lewis rats were randomized into eight groups. Six groups underwent heat shock priming of the periosteum of the calvaria at 42.5°C, two of them (n = 8) for 15 minutes, two (n = 8) for 25 minutes and two (n = 8) for 35 minutes. After 24 hours, a periosteal chamber was implanted into the heads of the animals of one of each of the two groups mentioned above. Microcirculation and inflammatory responses were studied repeatedly over a period of 14 days using intravital fluorescence microscopy. The expression of heat shock protein (HSP) 70 was examined by immunohistochemistry in three further groups 24 hours after a 15-minute (n = 5), a 25-minute (n = 5) or a 35-minute (n = 5) heat shock treatment. Two groups that did not undergo priming were used as controls. One control group (n = 8) was investigated by intravital microscopy and the other (n = 5) by immunohistochemistry.

**Results:**

During the entire observation period of 14 days, the periosteal chambers revealed physiological microcirculation of the periosteum of the calvaria without perfusion failures. A significant (p < 0.05) and continuous increase in functional capillary density was noted from day 5 to day 14 after 25-minute heat shock priming. Whereas a 15-minute exposure did not lead to an increase in functional capillary density, 35-minute priming caused a significant but reversible perfusion failure in capillaries. Non-perfused capillaries in the 35-minute treatment group were reperfused by day 10. Immunohistochemistry demonstrated an increase in cytoprotective HSP70 expression in the periosteum after a 15-minute and a 35-minute heat shock pretreatment when compared with the control group. The level of HSP70 expression that was measured in the periosteum after 25 minutes of treatment was significantly higher than the levels observed after 15 or 35 minutes of heat shock exposure.

**Conclusion:**

A few days after heat shock priming over an appropriate period of time, a continuous increase in functional capillary density is seen in the periosteum of the calvaria. This increase in perfusion appears to be the result of the induction of angiogenesis.

## Background

The periosteum is a highly vascularized membrane that covers bone. It consists of a fibro-elastic layer of tissue that is firmly attached to the bone surface. Although the bone cortex is the main beneficiary of the principal anatomical and physiological functions of the periosteal membrane, periosteal activity influences the behaviour of the entire bone [1]. Above all, the periosteum participates in osteogenesis, serves as an attachment site for muscles and ligaments and contributes to the blood supply to cortical bone [2,3]. Apart from its nutritive function, the periosteum has also a mechanical function and plays an important role in tissue repair. Following the surgical treatment of osseous defects, the periosteum is believed to be of paramount importance in the healing process [4,5]. In addition, the periosteum contributes substantially to bone growth. Capillary perfusion impairment or failure in the periosteum is reported to lead to disturbed bone growth especially in association with bone augmentation, bone distraction and cleft surgery [6]. A basic requirement for the preservation of periosteal functions is the presence of adequate blood flow in periosteal vessels. Especially in bone augmentation procedures, which are routinely performed prior to the insertion of dental implants, the presence of a well-vascularized recipient bed is essential for a successful outcome [6,7].

Exposure to a local sublethal heat shock is a possible method of increasing stress resistance. In response to heat shock priming, cells are believed to be more resistant against stress such as surgical trauma and reperfusion [8-10]. A heat shock leads to the expression of cytoprotective heat shock proteins (HSPs), which belong to a family of proteins that induce immunological cell activities, thermotolerance, buffering of expression of mutations and macrophage-mediated wound healing [11,12]. The upregulation of HSPs, however, induces not only intracellular but also extracellular processes [13-15]. In tissues, stress conditioning can reduce interstitial edema formation and improve perfusion as a result of blood flow upregulation [16]. Moreover, a relationship between heat shock proteins and angiogenic factors was detected in acute models [17,18]. Long-term effects on local microcirculation have not yet been investigated.

The objective of our study was therefore to study the effects of local heat shock priming on periosteal vascularization and inflammation using an in-vivo rat model. A further objective was to analyze the influence of the duration of exposure to a heat shock and the associated expression of HSPs using immunohistochemistry.

## Material and methods

### Animals

The study involved 52 male Lewis rats weighing between 300 to 330 g. All animals had ad libitum access to food and water. The rats were housed singly in cages. They were kept and the experiments were performed in accordance with the German Animal Protection Act. All animal procedures (dated 1 January 2007) had been approved by the Animal Protection Department in the Office of Consumer Protection and Food Safety of the State of Lower Saxony in Oldenburg.

### Heat shock priming

The periosteum of the calvaria of the anesthetized animals was exposed to a heat shock. For this purpose, the foreheads of the rats were shaved. Two copper tubes were placed on the shaved skin through which water was delivered. The heating procedure was standardized by exposing the periosteum of the calvaria to a temperature of 42.5°C for either 15, 25 or 35 minutes. During heat shock pretreatment, periosteal and systemic temperatures were measured using a modified thermometer.

### Chamber implantation

Intravital microscopy using a periosteal chamber has been previously described in detail.[19]

Briefly, the animals were anesthetized using an intraperitoneal injection of ketamine (Ketavet^®^, 100 mg per kg bodyweight, Parke-Davis, Germany) and xylazine (Rompun^®^, 5 mg per kg bodyweight, Bayer HealthCare, Germany). The periosteum was then exposed. Collagenous connective tissue was carefully excised using microsurgical instruments and a 3D microscope until the vascular layer of the periosteum was exposed. The frame of the chamber was placed on the periosteum and sutured to adjacent skin in such a way as to prevent drying (Ethicon Vicryl sutures 5-0, Johnson & Johnson, Germany). The cover glass was secured to the frame with a circlip.

### Intravital fluorescence microscopy

For intravital microscopy, the rats were again anesthetized with ketamine and xylazine immediately after the implantation of the chamber as well as on days 3, 5, 10, and 14 after surgery.

For high-resolution imaging of microcirculation, we injected fluorescein-isothiocyanate-labeled dextran (FITC-dextran, 150 000 MW, Sigma Chemicals, United States) for contrast enhancement by intravascular staining of blood plasma and rhodamine 6G (MG 476, Sigma Merck, Germany) for direct visualization of leukocytes. Immediately before each examination, 0.5 ml of FITC-dextran (150 mg/ml of 0.9% NaCl solution) and 0.5 ml of rhodamine 6G (1 mg/ml of 0.9% NaCl solution) were injected intravenously. Subsequently, the animals were immobilized on a special plexiglass table in such a way that the area to be examined was visible under the microscope and head movements caused by respiration were minimized.

Epi-illumination fluorescence microscopy was performed using a modified microscope (Zeiss microscope, Zeiss Fluoartic, Germany) at 20x magnification. A blue filter block (450- 490 nm) was used for the detection of FITC. A green filter block (530-560 nm) was used to visualize leukocytes labeled in vivo with rhodamine 6G. The microscopic images were recorded by a charge-coupled device (CCD) video camera (Pieper, FK-6990 IQ-S, Germany) and were transferred to a DVD recorder (LQ-MS 800, Panasonic, Osaka, Japan) for off-line evaluation. Images were recorded for 30 seconds at four different regions of interest (0.18 mm²). During microscopy, the body temperature of the animals was maintained at +36°C using a heating pad.

### Inflammatory parameters

We analyzed leukocyte-endothelial cell interaction in order to study inflammatory responses. For this purpose, leukocytes were classified as rolling or adherent cells depending on their interaction with endothelium. Adherent leukocytes were defined in each vessel segment as cells that did not move or detach from the endothelial lining within a specified observation period of 20 seconds. Results for adherent leukocytes are expressed as the number of cells per square millimeter of endothelial surface, calculated from the diameter and length of the vessel segment. Cylindrical vessel geometry was assumed. Rolling leukocytes were defined as cells that moved with a velocity less than two fifths of centerline velocity. Results for rolling leukocytes are expressed as the number of cells per minute that passed a defined reference point in a microvessel.

### Vascular perfusion

Microscopic images were analyzed off-line using image analysis software (CapImage, Zeintl, Heidelberg, Germany). Vessel diameter (μm) and functional microvessel density (mm/mm²) were determined for the assessment of microcirculatory parameters. Functional microvessel density was defined as the total length of blood cell-perfused microvessels per observation area and was expressed in mm/mm*^2^*. For the purpose of our analysis, we measured microvessel density at five observation areas at the various time points.

### Immunohistochemistry of heat shock protein (HSP) 70

After 24 hours of recovery from a 15-minute, a 25-minute or a 35-minute heat shock, specimens from the anesthetized animals were prepared for immunohistological analysis.

For the immunohistochemical detection of HSP70, paraffin-embedded specimens were cut into 5-μm-thick sections, deparaffinized with xylene and rehydrated. The sections were exposed to 2% normal goat serum (Dianova, Hamburg, Germany) diluted in phosphate-buffered saline (PBS, Biochrom, Berlin, Germany) to block non-specific binding. They were then incubated overnight at 4°C with a monoclonal mouse anti-HSP70 antibody (1:200, Acris, Hiddenhausen, Germany). Negative controls were not exposed to the primary antibody but to normal goat serum. After washing with PBS, the sections were incubated with biotinylated goat anti-mouse antibody (1:200, Dianova, Hamburg, Germany) and then with streptavidin-horseradish peroxidase complex (1:500, Dianova, Hamburg, Germany). Color was developed with aminoethylcarbazole (AEC) substrate (Vector, Burlingame, CA) at room temperature under microscopic examination. The sections were then washed with water, counterstained with hematoxylin, mounted using an aqueous mounting medium (Aquatex, Merck, Darmstadt, Germany) and examined by light microscopy (DM4000B Leica Mikrosysteme, Wetzlar, Germany).

The intensity of immunohistochemical staining for HSP70 was assessed using image analysis software (Analysis, Olympus Soft Imaging Solutions, Muenster, Germany). Briefly, digital micrograph data obtained for the immunohistochemical slides were imported from the microscope-mounted digital imaging system for the analysis of staining intensity. Regions of interest were defined. Staining intensity was measured in four samples from each animal and expressed as the percentage of positive pixels to total pixels.

### Study protocol

The animals were randomized into eight groups. In four groups (n = 20), the effects of heat shock exposure were analyzed after 15-minute (n = 5), 25-minute (n = 5) or 35-minute (n = 5) heat shock priming of the periosteum. The fourth group (n = 5) served as controls.

For intravital microscopy, 32 animals were placed into 4 groups (each with 8 animals). Periosteal chambers were inserted into all animals. Twenty-four hours prior to chamber implantation, three groups received local heat shock priming for 15, 25 or 35 minutes. Microcirculation and inflammation were studied immediately as well as 3, 5, 10 and 14 days after heat shock priming using intravital fluorescence microscopy.

### Statistical Analysis

Results are expressed as means ± SEM. Differences between groups were evaluated with a one-way analysis of variance (ANOVA). Differences within groups were analyzed by one-way repeated measures ANOVA. Student-Newman-Keuls or Dunn's post-hoc tests were used to isolate specific differences. A p-value <0.05 was considered significant.

## Results

During the entire observation period, the periosteal chamber enabled us to reliably view and monitor the periosteum covering the calvaria. No animal had macroscopic inflammation at the surgical site.

### Inflammatory response

**Figure 1:**
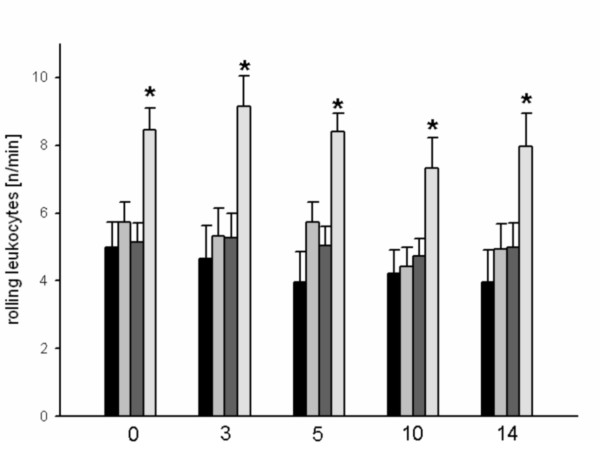
**Rolling leukocytes (n/min) at day 0, 3, 5 and 10 for controls (black bar) or after heat shock conditioning for 15 minutes (light grey bar), 25 minutes (dark grey bar) or 35 minutes (white bar), as assessed by intravital fluorescence microscopy and computer-assisted off-line analysis**. There were significant higher rolling leukocytes detectable after 35 minutes of heat shock conditioning over the entire observation time (* p < 0.05 vs. control group).

**Figure 2:**
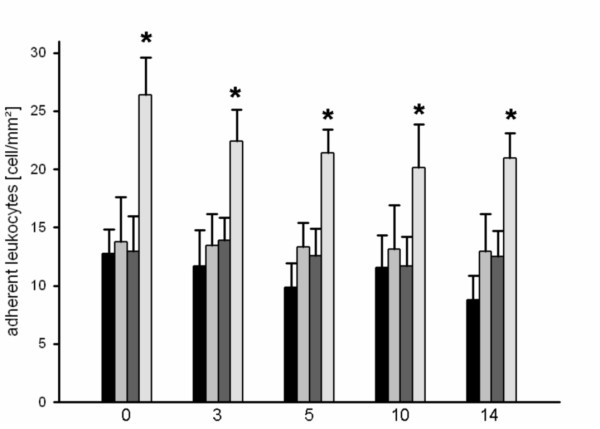
**Adherent leukocytes at day 0, 3, 5 and 10 for controls (black bar) or after heat shock conditioning for 15 minutes (light grey bar), 25 minutes (dark grey bar) or 35 minutes (white bar), as assessed by intravital fluorescence microscopy and computer-assisted off-line analysis**. There were significant higher adherent leukocytes detectable after 35 minutes of heat shock priming over the entire observation time (* p < 0.05 vs. control group).

Compared with the control group, the groups of animals that underwent 15-minute or 25-minute heat shock priming showed slightly elevated numbers of rolling leukocytes (Figure 1). By contrast, a 35-minute heat shock induced a marked inflammatory response as indicated by a significantly higher number of rolling leukocytes during the entire observation period. The control group and the group that received 25-minute heat shock priming showed constantly low numbers of adherent leukocytes during the observation period. In the group of animals that were exposed to a 15-minute heat shock, the number of adherent leukocytes was slightly increased until day 9 after surgery and then declined to levels similar to those of the control group. When compared with all other groups, the group with a 35-minute treatment showed a significant increase in the number of adherent leukocytes during the entire observation period (Figure 2).

### Microcirculatory parameters

Intravital fluorescence microscopy allows us to study the network of microvessels that run parallel to the tissue surface over a period of 14 days. Vessels that are oriented perpendicular to the tissue surface and connect either to subcutaneous tissue or underlying bone cannot be identified. An examination of the periosteum revealed that the majority of capillaries were arranged in a single layer.

**Figure 3:**
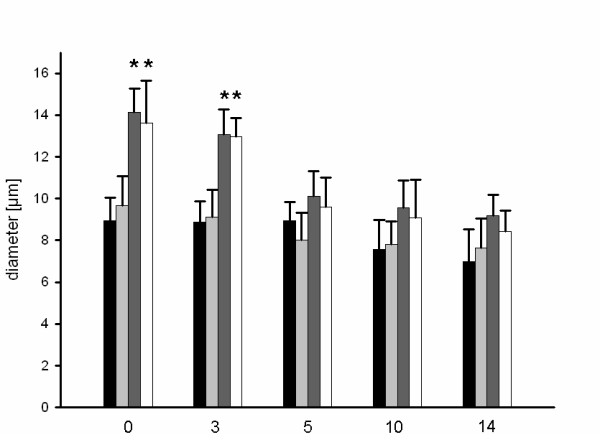
**Microvessel diameter (μm) at day 0, 3, 5 and 10 for controls (black bar) or after heat shock conditioning for 15 minutes (light grey bar), 25 minutes (dark grey bar) or 35 minutes (white bar), as assessed by intravital fluorescence microscopy and computer-assisted off-line analysis**. There was a significant higher microvessel diameter up to day 5 detectable after 25 and 35 minutes of heat shock conditioning (* p < 0.05 vs. control group).

A comparison between the control group and the group with 15-minute heat shock priming showed no differences in vessel diameters (Figure 3). By contrast, a significant increase in vessel diameters was found over a period of five days in those groups that underwent heat shock treatment for 25 or 35 minutes. After day 5, all groups showed similar results.

**Figure 4:**
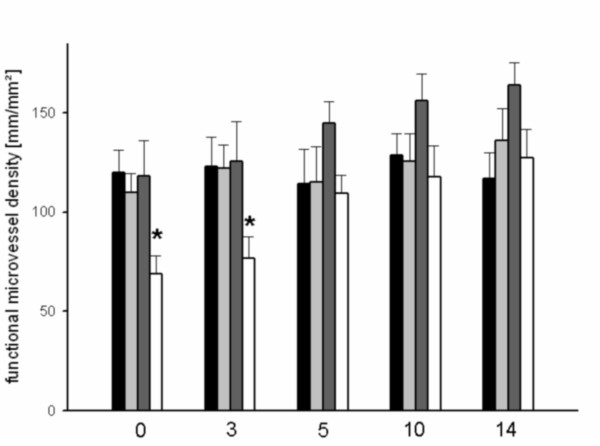
**Functional microvessel density at day 0, 3, 5 and 10 for controls (black bar) or after heat shock conditioning for 15 minutes (light grey bar), 25 minutes (dark grey bar) or 35 minutes (white bar), as assessed by intravital fluorescence microscopy and computer-assisted off-line analysis**. There was significant lower functional microvessel density detectable up to day 5 after 35 minutes of heat shock conditioning.

The control group and the group that received 15-minute heat shock priming showed similar results for functional microvessel density (Figure 4). These results were almost constant over the entire observation period. In the group with a 25-minute heat shock pretreatment, functional capillary density continuously increased from day 5 to the end of the observation period (Figure 4). After day 5 a significant increase (p < 0.05) in microvessel density was observed compared to day 0 after heat shock conditioning. Both buds and sprouts were identified morphologically. By contrast, a significantly lower functional microvessel density was detected until day 5 in the group that underwent 35 minutes of heat shock priming. Non-perfused microvessels were detected until day 5 after heat shock exposure. From day 5 onwards, reperfusion of individual non-perfused capillaries was observed. Reperfusion of all non-perfused capillaries was completed on day 10. All groups showed similar results for red blood cell velocity in perfused microvessels.

### Histology

Regardless of the duration of heat shock priming, there were no morphological differences in the structure of the periosteum between the groups with and without heat shock exposure. Rather, the periosteum was structurally intact and similar in heat-shocked and control animals.

**Figure 5:**
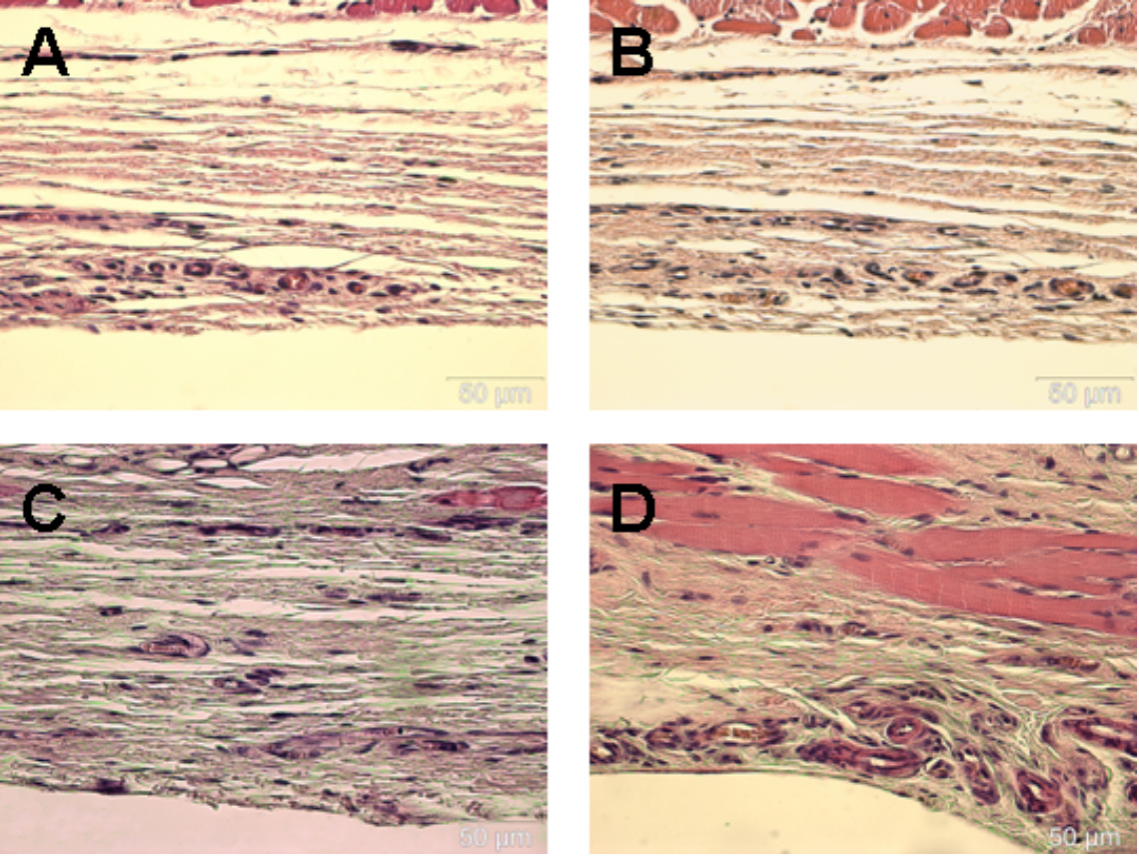
**Histological sections after staining with a monoclonal mouse anti-HSP70 antibody revealing in the control group HSP70 expression near the detection limit (A), whereas slight detection after heat shock conditioning after 15 and 35 minutes (B and D) are shown**. The highest expression was detectable after 25 minutes (C) of heat shock conditioning.

An analysis of immunohistological specimens from the control group (0.06% ± 0.04) revealed a very low level of HSP70 expression near the detection limit. Twenty-four hours after heat shock treatment, different levels of HSP70 expression were induced in the periosteum (Figure 5). Irrespective of the duration of priming, the levels of HSP70 expression in the periosteum were significantly higher in the groups of heat-shocked animals than in the control group. It was particularly noteworthy that the level of HSP70 expression was higher after 25 minutes (6.38% ± 09) of heat shock priming than after 15 (1.42% ± 0.4) or 35 (1,18% ± 0.6) minutes of exposure.

## Discussion

After 15 minutes of heat shock priming, there were only minor changes in the microcirculatory perfusion of the periosteum. After 35 minutes of pretreatment, there was a temporary decrease in perfused capillary density. After 25 minutes of heat shock treatment, however, minor signs of local inflammation and a constant increase in functional capillary density were observed until day 14 after the application of a heat shock.

Several methods such as laser Doppler flowmetry or polarographic oximetry can be used for examining perfusion of different tissues in vivo [2,20,21]. These methods, however, have the disadvantage that they can visualize blood flow only indirectly. It is therefore impossible to measure blood perfusion of individual microvessels using these techniques. By contrast, intravital microscopy allows the perfusion of microvessels to be examined over an extended period of time [21,22].

The periosteum of the calvaria is difficult to examine on account of its anatomical location and physiological adherence to underlying bone. For this reason, only a few methods are available for investigating the microcirculation of the periosteum of the calvaria in vivo. Previous studies of the periosteum have therefore been based on acute examinations especially of histological specimens [23,24]. The chamber model presented here allowed us to evaluate the periosteum in vivo repeatedly over several days [19]. As expected, the control group did not show any significant changes in microcirculatory perfusion or local signs of inflammation during the observation period. Likewise, there were hardly any inflammatory tissue responses to 15 minutes of heat shock exposure. The animals that received a 15-minute heat shock treatment showed results similar to those obtained for control animals not only in terms of inflammatory tissue responses but also in terms of vessel diameter. Fifteen minutes of heat shock priming induced only minor changes in capillary blood flow and functional capillary density. In addition, this animal group showed a low level of HSP70 expression near the detection limit. This means that heat shock priming for a period of 15 minutes has only a minor effect on microcirculation of the periosteum.

By contrast, 35 minutes of heat shock priming caused a marked temporary increase in local inflammation as well as a temporary dilation of perfused vessels and an increased blood flow. Thirty-five minutes of heat shock pretreatment was, however, associated with significant perfusion failure (about one third of total microvascular perfusion) during the first few days. It is likely that the application of heat caused endothelial cell damage at the vascular endothelium and induced an increased inflammatory tissue response [25]. This supports the findings reported by Menger et al., who detected an endothelin (ET)-1-mediated local inflammatory response after microvascular constriction following heat application [26]. This led to perfusion failures in microvessels [27]. The level of HSP70 expression after 35-minute heat shock priming was similar to that noted after a 15-minute exposure. This results suggests that 35 minutes of heat shock treatment is an excessively strong thermal stimulus and thus prevents an increased expression of HSP70.

By contrast, 25-minute heat shock priming induced mild inflammatory responses similar to those seen in the control group and after a 15-minute exposure. A similar effect was described by Ruecker et al [15] in their study on osteomyocutaneous flaps and is likely to be the result of the antioxidative action of biliverdin, which prevents the up-regulation of leukocytic adhesion molecules such as ICAM-1 [28].

In addition, a 25-minute heat shock treatment was not associated with perfusion failure. Compared with the control group, the animals in the 25-minute heat shock group showed a temporary dilation of vessels and an increase in functional capillary density. Both buds and sprouts were identified from day 5 onwards. Processes such as proliferation, sprouting and remodeling of existing endothelial cells lead to the generation of new microvessels. Since there was no perfusion failure in the control group and 25-minute heat shock priming was associated with the formation of buds and sprouts, the increase in functional capillary density appears to be the result of the formation of new vessels. This form of aniogenesis led to a significant increase in functional capillary density until day 14.

Angiogenesis is induced by different angiogenic factors such as transforming growth factor (TGF) or vascular endothelial growth factor (VEGF) [29,30]. Gong et al. demonstrated a relationship between VEGF and HSP70 in association with whole-body hyperthermia. It is generally known that the exposure of tissue to sublethal stress can lead to the formation of heat shock proteins [31,32]. The study presented here shows that the in-vivo expression of HSP70 depends not only on temperature but also on the duration of thermal pretreatment of the periosteum. This finding extends the current knowledge of in-vitro HSP70 expression [33]. We were able to demonstrate that HSP70 expression is influenced by the duration of heat shock priming. The highest level of expression was observed after 25 minutes of local heat shock priming and is correlated with an increase in functional capillary density from day 5 onwards. This shows that a heat shock induces a significant increase in periosteal perfusion. For this reason, heat shock exposure is an effective form of pretreatment before procedures that compromise periosteal microcirculation such as distraction or bone augmentation.

## Competing interests

The authors declare that they have no competing interests.

## Authors' contributions

MR, CS, MRU, PS, HE, HK, DL and NCG conceived of the study and participated in its design and coordination. MW and CS drafted the manuscript. All authors read and approved the final manuscript.

## Funding

The article processing charges are funded by the Deutsche Forschungsgemeinschaft (DFG), "Open Acess Publizieren".
